# Exploring trajectories of acute kidney injury in the intensive care unit: a population-based cohort study

**DOI:** 10.1038/s41598-025-11587-6

**Published:** 2025-09-26

**Authors:** Ding-yuan Wan, Xin-yao Luo, Min He, Zhong-wei Zhang

**Affiliations:** 1 West China School of Medicine, West China Hospital, Sichuan University, Chengdu, China; 2 Department of Critical Care Medicine, West China Hospital, Sichuan University, Chengdu, China; 3 Department of Nephrology, West China Hospital, Sichuan University, Chengdu, China; 4 Department of Intensive Care Medicine, West China Hospital, Sichuan University, 37 Guo Xue Xiang, Chengdu, 610041 China

**Keywords:** Group-based trajectory modeling, Endothelial activation and stress index score, Acute kidney injury, Subphenotyping, Prognostic markers, Prognostic markers, Acute kidney injury, Nephrology, Risk factors

## Abstract

Acute kidney injury (AKI) represents a complex disorder characterized by distinct subphenotypes with varied clinical presentations and prognoses. Categorizing these subphenotypes may facilitate standardization of research cohorts and optimization of therapeutic strategies. The endothelial activation and stress index (EASIX) quantifies thrombotic microangiopathy severity, a pathophysiological hallmark of AKI. Consequently, we utilized EASIX trajectory analysis to identify AKI subphenotypes. AKI patients were identified from the eICU Collaborative Research Database to develop a group-based trajectory model. EASIX scores recorded during the initial seven ICU days were utilized for trajectory modeling. Patients were stratified into distinct subgroups according to the best model. Variable selection was performed using LASSO regression, followed by multivariate Cox regression analyses to calculate hazard ratios (HRs) across the identified subgroups. An independent validation cohort comprised patients from the central ICU of West China Hospital (WCH). The study’s primary endpoints included all-cause in-ICU and in-hospital mortality across the identified subphenotypes. The final analysis included 317 patients from the eICU database and 58 patients from WCH. Based on the EASIX trajectories derived from the first seven ICU days, we identified two distinct subphenotypes: a “Stably High” (SH) group and a “Decreasing” (D) group. Compared to the D group, the SH group demonstrated significantly higher mortality risk, with an HR of 2.26 (95% CI 1.14–4.26, *p* = 0.018) for ICU mortality and 1.85 (95% CI 1.03–3.29, *p* = 0.038) for 30-day in-hospital mortality. These findings were replicated in the WCH validation cohort. This study identified and validated two distinct AKI subphenotypes through EASIX trajectory analysis, demonstrating significant heterogeneity in clinical characteristics, laboratory findings, comorbidities, and outcomes between these groups. Future research may focus on early subphenotype prediction, differential treatment responses, and molecular mechanisms driving inter-group variation.

## Backgrounds

Acute kidney injury (AKI) is a clinically and biologically heterogeneous syndrome that affects nearly one-third of intensive care unit (ICU) patients^1^. While current KDIGO staging criteria classify AKI severity based on clinical manifestations, this approach fails to capture the diverse pathophysiological processes that underlie phenotypically similar cases. Such heterogeneity, manifested through varying molecular mechanisms, differential treatment responses, and distinct clinical trajectories, likely contributes to inconsistent therapeutic outcomes and challenges in developing targeted interventions. Improved subphenotyping could address these limitations by enabling more precise risk stratification, mechanistic investigation, and therapeutic development.

Refining AKI classification beyond serum creatinine criteria could enhance clinical understanding of disease progression and facilitate early treatment response assessment. Emerging evidence across AKI, oncology, and critical care medicine suggests that subphenotype identification provides pathophysiological insights to guide precision therapies^2–5^. For example, Calfee et al. demonstrated that direct versus indirect lung injury in acute respiratory distress syndrome displays divergent biomarker patterns, reflecting distinct biological mechanisms^2^. Similarly, Bhatraju et al. established that AKI subphenotypes defined by creatinine trajectories predict mortality differentials^3^. Stratifying AKI based on trajectory enables the consideration of the patient’s initial reaction to early therapeutic interventions.

Recently, the endothelial activation and stress index (EASIX) has emerged as a promising biomarker for assessing endothelial damage^6^. It is calculated from routine laboratory parameters: the product of serum lactate dehydrogenase levels (U/L) and creatinine levels (mg/dL) divided by the platelet count (10^9/L). Endothelial dysfunction, marked by impaired vascular regulation along with increased inflammatory and procoagulant activity, represents a key mechanism in AKI development^7,8^. Analysis of EASIX trajectories could enable more precise identification of AKI subphenotypes, offering enhanced risk stratification and the potential to define more clinically homogeneous patient groups compared to current classification methods.

Our study employed EASIX trajectory analysis to characterize AKI subphenotypes. We proposed that stratifying AKI patients based on EASIX score dynamics during the first seven days of ICU admission would effectively differentiate subgroups with distinct mortality risks.

## Methods

### Data sources

The eICU Collaborative Research Database, containing de-identified clinical data from 139,361 ICU admissions across U.S. hospitals (2014–2015), served as the training cohort for this study (Authorization No. 60143064). For external validation, we utilized electronic health records from the central ICU at West China Hospital, a premier tertiary medical center in Southwest China, which included demographic information, vital signs, and laboratory results. The Biomedical Research Ethics Committee of West China Hospital approved this retrospective analysis (No.20211687) and waived informed consent due to the anonymized nature of the data.

### Inclusion and exclusion criteria

Patients are included in this study provided that they (1) were more than 18 years old, (2) had enough data to compute EASIX scores, (3) stayed in the ICU for more than 7 days, and (4) met KDIGO criteria for AKI upon ICU admission^9^; however, these patients were not excluded due to (1) having fewer than 2 records of EASIX scores within the first seven days after ICU and (2) having more than 5% of variables missing.

### Covariables and outcomes

The analysis incorporated demographic variables (age, sex, weight, and comorbidities such as hypertension, atrial fibrillation, heart failure, malignancy, and chronic respiratory disease), vital signs (heart rate, respiratory rate, mean arterial pressure, temperature, and urine output), laboratory parameters (including electrolyte levels, hematologic indices, liver enzymes, and metabolic markers), and clinical scores (Glasgow Coma Scale and APACHE) recorded at ICU admission. EASIX scores were calculated using the standard formula: (lactate dehydrogenase [U/L] × creatinine [mg/dL])/platelet count [10⁹/L]. The primary endpoints assessed all-cause in-ICU and in-hospital mortality the identified subphenotypes.

### Statistical analysis

The group-based trajectory model (GBTM), a specialized latent class mixed model, identifies distinct clinical trajectories within patient populations. In this study, we applied GBTM to analyze EASIX score patterns during the first 7 days of ICU stay in AKI patients. The class-defining variables used in the GBTM are based on the overall performance of the identified trajectories and the background of clinical knowledge. Age, sodium, potassium, white blood cell count, hematocrit, pH, bicarbonate, heart rate, respiratory rate, mean arterial blood pressure and temperature were selected as the final class-defining variables and were grouped into different subgroups according to breakpoints used in the APACHE scoring system (for example, the variable age was grouped into five different groups: age < 45, 45 ≤ age < 55, 55 ≤ age < 65 y, 65y ≤ age < 75 y, and 75y ≤ age). To ensure robust model fitting, we performed extensive computational testing by generating 500 GBTM iterations across 1–6 latent class solutions, with each configuration replicated 100 times to avoid local optimum convergence. The final model selection balanced statistical performance with clinical interpretability of the derived trajectories. The optimal model was selected through comprehensive evaluation of multiple statistical metrics including log likelihood, Bayesian information criterion (BIC), group membership percentage, and average posterior probability combined with clinical expertise. We classified eICU patients into distinct subgroups using the optimal model and analyzed inter-group differences in crude mortality rates. Survival analysis included log-rank testing for mortality risk differences across subphenotypes and multivariable Cox regression to calculate adjusted hazard ratios. Covariates for regression models were selected through LASSO regression with clinical expert validation. We externally validated findings by applying identical GBTM parameters to the WCH cohort, repeating all survival analyses using the same statistical methods. Statistical significance was defined as *p* < 0.05.

Continuous variables with normal distribution were expressed as mean ± standard deviation, while non-normally distributed data were reported as median (interquartile range). Categorical variables were summarized as counts (percentages). Group comparisons employed appropriate statistical tests: Student’s t-tests for normally distributed continuous variables, Wilcoxon rank-sum tests for non-parametric data, and chi-square tests for categorical variables. Missing data were addressed using multiple imputation by chained equations. All analyses were performed in R (version 4.1) utilizing the ‘mice’, ‘lcmm’, ‘survival’, ‘survminer’, and ‘glmnet’ packages. All methods were performed in accordance with relevant guidelines and regulations.

## Results

### Patient characteristics

**Fig. 1:**
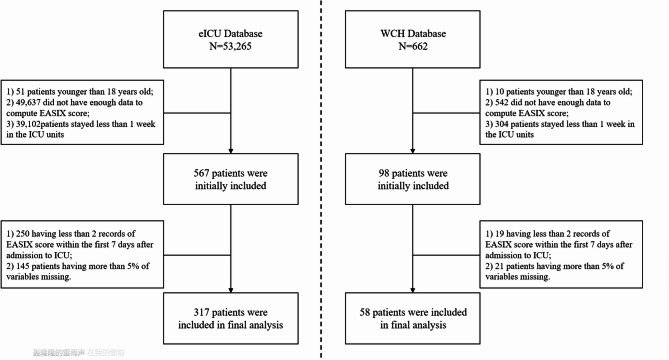
Study flowchart with patient inclusion/exclusion criteria. ICU, intensive care unit; WCH, West China Hospital.

The analysis initially identified 567 adult AKI patients from the eICU database, with 317 meeting all criteria for model development (Fig. 1). The cohort had a median age of 62 years (IQR 48–71), with 57.4% (*n* = 182) male and 75.7% (*n* = 240) white patients. At ICU admission, the median APACHE score was 81 (IQR 64–102). The cohort demonstrated the following AKI severity distribution per KDIGO criteria: stage 3 (*n* = 124, 39.1%) of cases, followed by stage 2 (*n* = 102, 32.2%), and stage 1 (*n* = 91, 28.7%). Additional baseline characteristics are presented in Table 1.

**Table 1:** Baseline characteristics of the overall population, Decreasing, and stably high groups in eICU database. AST: aspartate aminotransferase; ALT: Alanine aminotransferase; WBC: white blood cell count; RBC: red blood cell count; BUN: blood Urea nitrogen; LDH: lactate dehydrogenase; MAP: mean atrial pressure; GCS: Glasgow coma scale; CKD: chronic kidney disease7.

	Overall	Decreasing			Stably High	*p*
*N*	317	131			186	
**Age**	62.00 [48.00, 71.00]	63.00 [49.50, 73.00]			61.50 [48.00, 70.00]	0.187
**Sex (%)**						1
Female	135 (42.6)	56 (42.7)			79 (42.5)	
Male	182 (57.4)	75 (57.3)			107 (57.5)	
**Ethnicity (%)**						0.805
White	240 (75.7)	99 (75.6)			141 (75.8)	
Black	49 (15.5)	19 (14.5)			30 (16.1)	
Other	28 (8.8)	13 (9.9)			15 (8.1)	
**Hemoglobin**	9.45 [8.24, 11.25]	9.72 [8.43, 11.53]			9.08 [8.15, 11.06]	0.037
**WBC**	11.95 [8.38, 17.70]	13.25 [9.52, 17.68]			11.24 [7.50, 17.70]	0.016
**RBC**	3.20 [2.78, 3.80]	3.42 [2.90, 3.91]			3.07 [2.72, 3.71]	0.005
**Platelets**	144.00 [81.00, 224.00]	209.00 [149.00, 287.50]			100.50 [61.25, 163.75]	< 0.001
**AST**	53.75 [27.00, 115.00]	36.00 [22.62, 68.00]			66.50 [33.62, 174.38]	< 0.001
**ALT**	34.12 [19.88, 73.62]	27.25 [16.00, 53.25]			42.00 [22.00, 90.00]	0.001
**Creatinine**	1.50 [0.90, 2.55]	1.00 [0.68, 1.56]			2.00 [1.29, 3.10]	< 0.001
**BUN**	31.00 [19.04, 47.17]	21.50 [15.40, 33.38]			37.25 [24.12, 53.00]	< 0.001
**LDH**	321.00 [235.00, 496.00]	269.00 [198.00, 351.00]			380.00 [270.00, 610.50]	< 0.001
**Spo2**	97.00 [95.00, 99.00]	98.00 [96.00, 99.00]			97.00 [95.00, 98.00]	0.021
**PH**	7.36 [7.32, 7.41]	7.38 [7.33, 7.43]			7.34 [7.31, 7.40]	< 0.001
**Lactate**	1.86 [1.20, 2.88]	1.61 [1.00, 2.19]			2.20 [1.39, 3.63]	< 0.001
**HCO3**	21.10 [18.69, 24.40]	21.85 [19.99, 26.04]			20.14 [17.83, 23.44]	< 0.001
**Sodium**	139.25 [135.50, 142.25]	139.62 [136.92, 142.50]			138.77 [135.02, 141.73]	0.078
**Potassium**	4.10 [3.77, 4.44]	4.02 [3.72, 4.26]			4.18 [3.80, 4.57]	0.005
**Heartrate**	94.00 [80.00, 107.00]	91.00 [79.00, 104.50]			97.00 [83.00, 109.00]	0.062
**Respiratory rate**	21.00 [17.00, 25.00]	19.00 [16.00, 23.00]			22.00 [18.00, 25.75]	< 0.001
**Temperature**	37.00 [37.00, 37.00]	37.00 [37.00, 37.00]			37.00 [37.00, 37.00]	0.103
**MAP**	77.33 [71.67, 85.33]	80.00 [73.17, 87.67]			75.33 [71.00, 82.92]	< 0.001
**ICU Stay**	11.08 [8.61, 16.10]		10.92 [8.42, 16.23]	11.22 [8.71, 16.04]		0.567
**Hospital Stay**	19.09 [13.21, 26.66]		17.46 [12.30, 25.94]	20.03 [13.78, 27.84]		0.069
**Clinical Score**						
GCS	8.00 [6.00, 12.00]	7.00 [4.00, 11.00]			8.00 [6.00, 12.00]	0.086
APACHE	81.00 [64.00, 102.00]	77.00 [56.00, 96.50]			83.50 [68.00, 104.75]	0.010
**Aki grade (%)**						< 0.001
1	91 (28.7)	51 (38.9)			40 (21.5)	
2	102 (32.2)	50 (38.2)			52 (28.0)	
3	124 (39.1)	30 (22.9)			94 (50.5)	
**Comorbidity (%)**						
Heart failure	35 (11.0)	13 (9.9)			22 (11.8)	0.726
Hypertension	39 (12.3)	15 (11.5)			24 (12.9)	0.83
Diabetes	13 (4.1)	6 (4.6)			7 (3.8)	0.941
CKD	51 (16.1)	13 (9.9)			38 (20.4)	0.019
Shock	65 (20.5)	14 (10.7)			51 (27.4)	< 0.001
Sepsis	109 (34.4)	31 (23.7)			78 (41.9)	0.001

### Subphenotypes of AKI patients

**Fig. 2:**
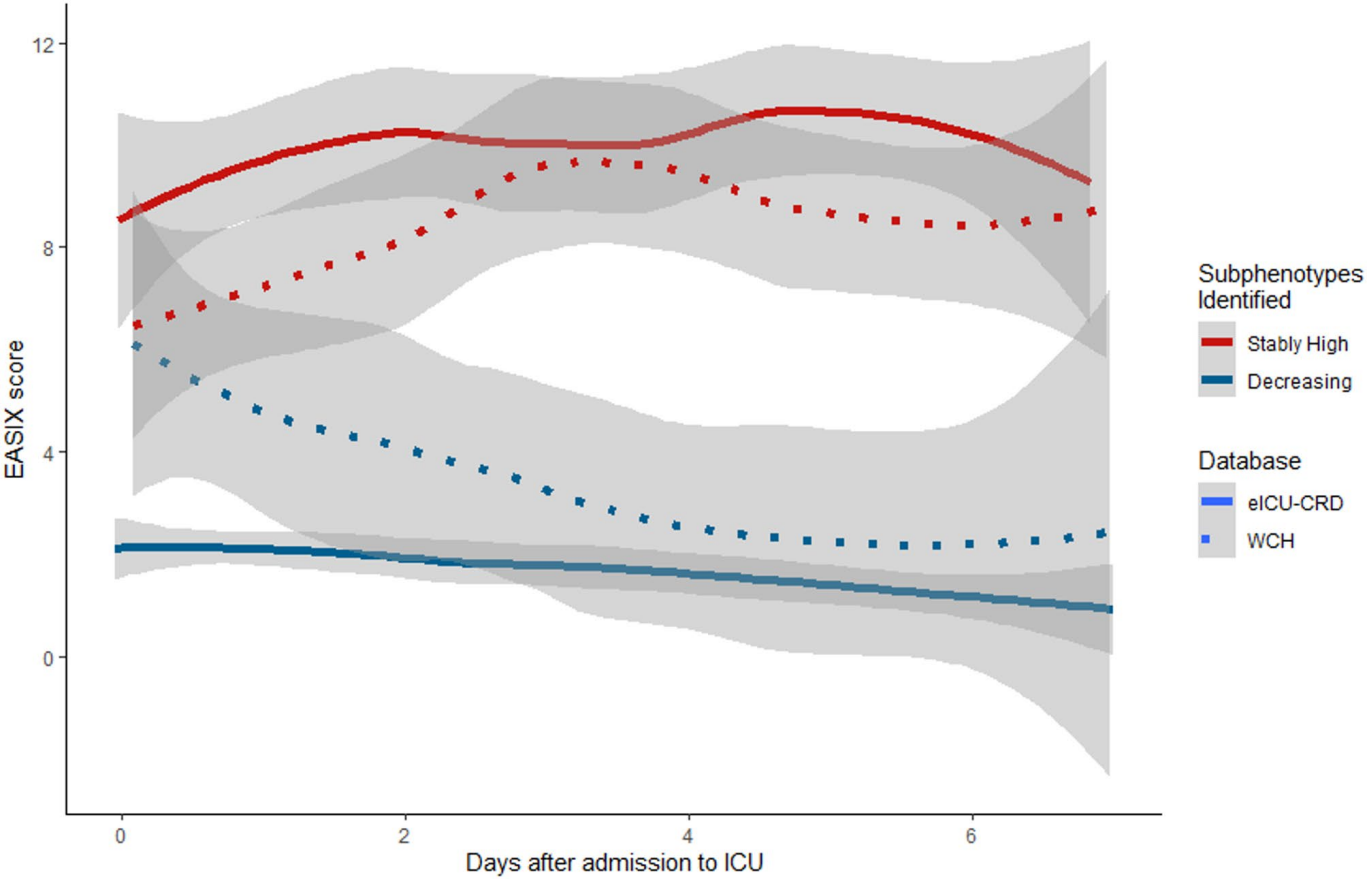
Endothelial activation and stress index (EASIX) trajectories of both subphenotypes in eICU and West China Hospital (WCH) Database. The solid lines represented EASIX trajectories from the eICU database, and the dotted lines represented those from the WCH database. The Stably High and Decreasing groups are represented in red and blue, respectively.

We constructed a total of 600 GBTMs and selected optimal models for each latent group configuration. The BIC, average posterior probability, and number of subjects of each configuration were listed in Table 2. The model of 2 latent groups was considered the best model based on the BIC and “elbow” criteria. The EASIX trajectories of two different groups, namely, the “stably high” (SH) and “decreasing” (D) groups, are shown in Fig. 2. A total of 186 patients were classified into the SH group, and the other 131 patients were classified into the D group. While demographic characteristics were comparable between groups (SH vs. D, age: 61.5 vs. 63 years, *p* > 0.05; male sex: 57.5% vs. 57.3%, *p* > 0.05), the SH group demonstrated significantly greater disease severity. This was evidenced by: (1) higher prevalence of stage 3 AKI (SH vs. D, 50.5% vs. 22.9%, *p* < 0.001), (2) increased rates of comorbidities including chronic renal disease (SH vs. D, 20.4% vs. 9.9%, *p* = 0.019), shock (SH vs. D, 27.4% vs. 10.7%, *p* < 0.001), and sepsis (SH vs. D, 41.9% vs. 23.7%, *p* = 0.001). Biochemical markers (serum lactate, potassium, and creatinine) were also significantly elevated in the SH group (Table 1).

**Table 2:** Fit indices for latent class trajectory models for endothelial activation and stress index. 1. Number of group members/average of posterior probabilities.

Group Number	Log Likelihood	BIC	Class1	Class2	Class3	Class4	Class5	Class6
1	−1873.376	3812.768	317/100%^1^	--	--	--	--	--
2	−1471.494	3253.968	131/92.48%	186/94.32%	--	--	--	--
3	−1438.880	3436.373	135/92.68%	136/92.00%	46/96.02%	--	--	--
4	−1410.252	3626.750	82/92.19%	44/97.59%	49/88.29%	142/90.74%	--	--
5	−1387.434	3828.747	66/94.40%	52/91.40%	63/91.30%	60/92.43%	76/91.90%	--
6	−1353.682	4008.877	64/95.95%	23/99.20%	41/94.05%	66/96.55%	52/93.58%	71/96.11%

### Survival outcomes

**Fig. 3:**
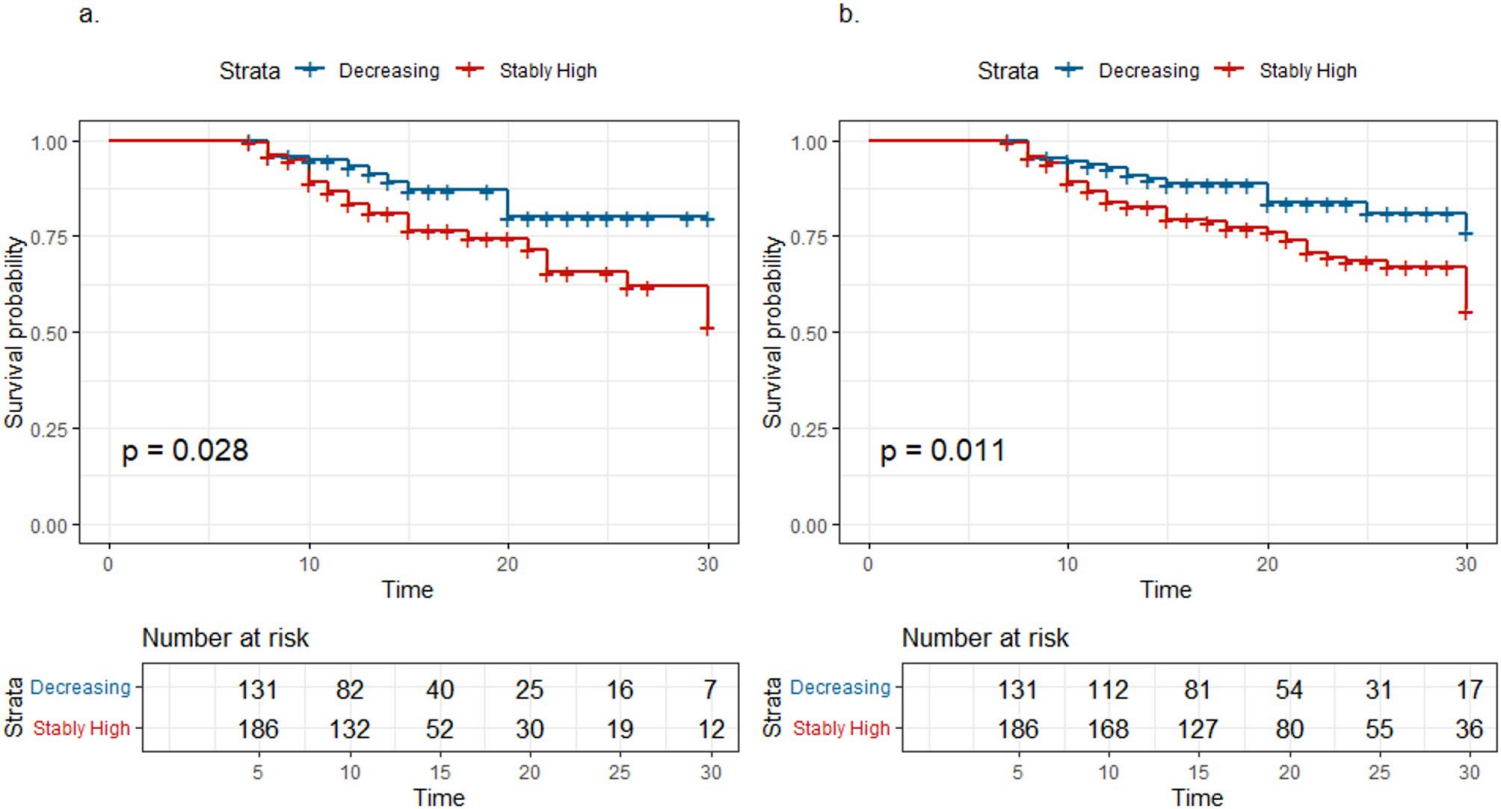
K‒M survival plot of patients with acute kidney injury in the eICU. (**a**) Survival plot of in-ICU mortality in a 30-day period; (**b**) survival plot of in-hospital mortality in a 30-day period.

**Fig. 4:**
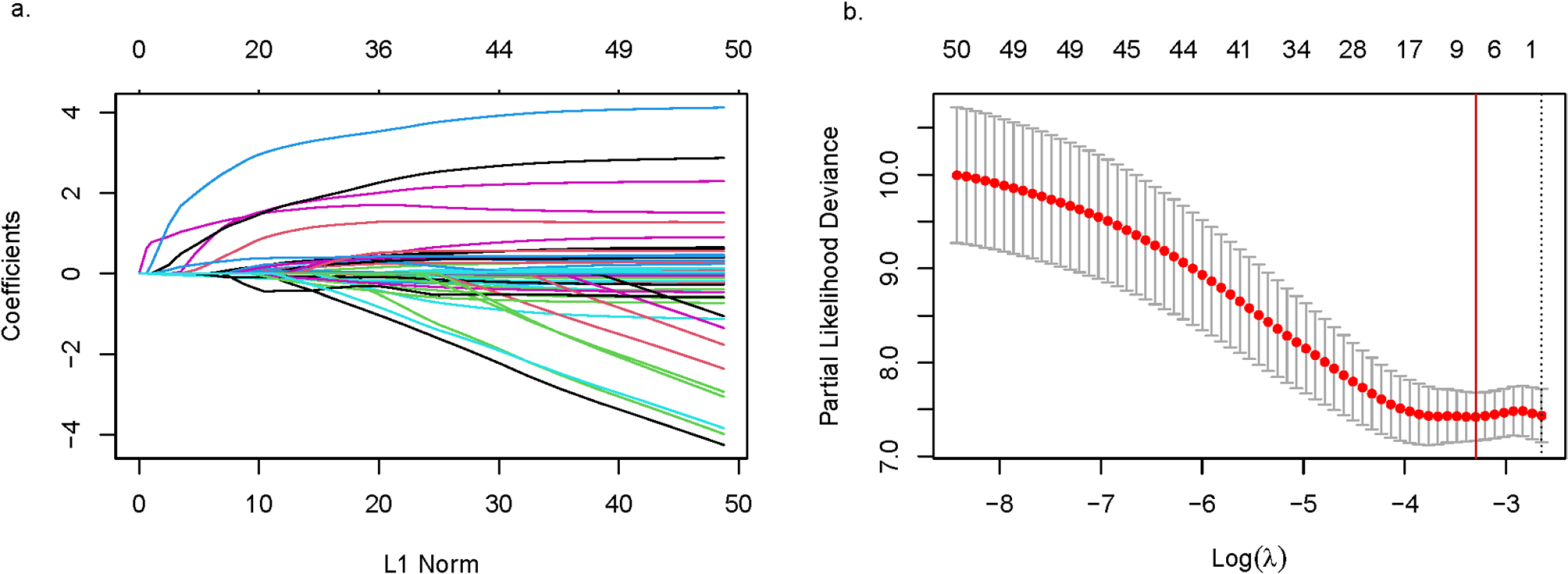
LASSO regression model. (**a**) LASSO coefficient profiles of the 24 candidate variables with up to 50 coefficients. Each curve represents a coefficient. (**b**) Cross-validation to select the optimal tuning parameter (λ). The red vertical line crosses over the optimal log λ.

Log-rank analysis demonstrated significant differences in both ICU and hospital mortality risks between subphenotypes throughout the 30-day observation period (Fig. 3). To evaluate the hazard ratio (HR) of the SH group compared to the D group for in-ICU and in-hospital mortality, a multivariate Cox proportional hazards analysis was conducted. Due to the limited number of events (47 and 72 deaths during ICU stay and hospitalization, respectively), an extensive selection of confounders was not feasible. LASSO regression (Fig. 4) was employed to determine the optimal number and covariates for inclusion. The full multivariate Cox model incorporated nine variables: subphenotype group, KDIGO AKI stage, age, sex, chronic renal disease status, shock status, sepsis status, white blood cell count, and pH, whereas the reduced model retained only subphenotype group, KDIGO AKI stage, age, and sex.

According to the full model, the estimated HR for 30-day in-ICU mortality in the SH group versus the D group was 2.26 (95% CI 1.14–4.26, *p* = 0.018, D group as reference). Notably, KDIGO AKI grade did not demonstrate a statistically significant association, with an HR of 0.60 (95% CI 0.31–1.15, *p* > 0.05) for grade 2 and 0.66 (95% CI 0.37–1.17, *p* > 0.05) for grade 3 (grade 1 AKI as reference) (Table 3). For 30-day in-hospital mortality, the HR was 1.85 (95% CI 1.04–3.29, *p* = 0.038, D group as reference), with KDIGO AKI grades remained non-significant, consistent with prior findings. Kaplan-Meier survival curves for in-ICU and in-hospital mortality are presented in Fig. 3a and b, respectively.

**Table 3:** Estimated hazard ratio of subphenotypes and KDIGO AKI group. Full model includes subphenotype groups, KDIGO AKI (Kidney disease: improving global outcomes acute kidney injury) group, age, gender, chronic renal disease, shock, sepsis, white blood cell count, and ph; and reduced model only includes subphenotype groups, KDIGO AKI group, age, and gender; WCH: West China hospital.

Variable	eICU				WCH	
	in-ICU		in-Hospital		in-ICU	
	Reduced model	Full model	Reduced model	Full model	Reduced model	Full model
**Subphenotypes**						
Decreasing	--	--	--	--	--	--
Stably High	2.12 (95%CI 1.07–4.19)	2.26 (95%CI 1.14–4.26)	1.89 (95%CI 1.09–3.27)	1.85 (95%CI 1.03–3.29)	2.03(95%CI 0.87–4.75)	1.52 (95%CI 0.54–4.23)
**KDIGO AKI grade**						
1	--	--	--	--	--	--
2	0.72 (95%CI 0.32–1.58)	0.60 (95%CI 0.31–1.15)	1.04 (95%CI 0.54-2.00)	0.93 (95%CI 0.48–1.80)	1.93 (95%CI 0.66–5.63)	1.90 (95%CI 0.47–7.7)
3	0.77 (95%CI 0.38–1.58)	0.66 (95%CI 0.37–1.17)	1.12 (95%CI 0.61–2.03)	1.10 (95%CI 0.60–2.05)	1.74 (95%CI 0.70–4.37)	1.72 (95%CI 0.53–5.63)

### External validation

**Fig. 5:**
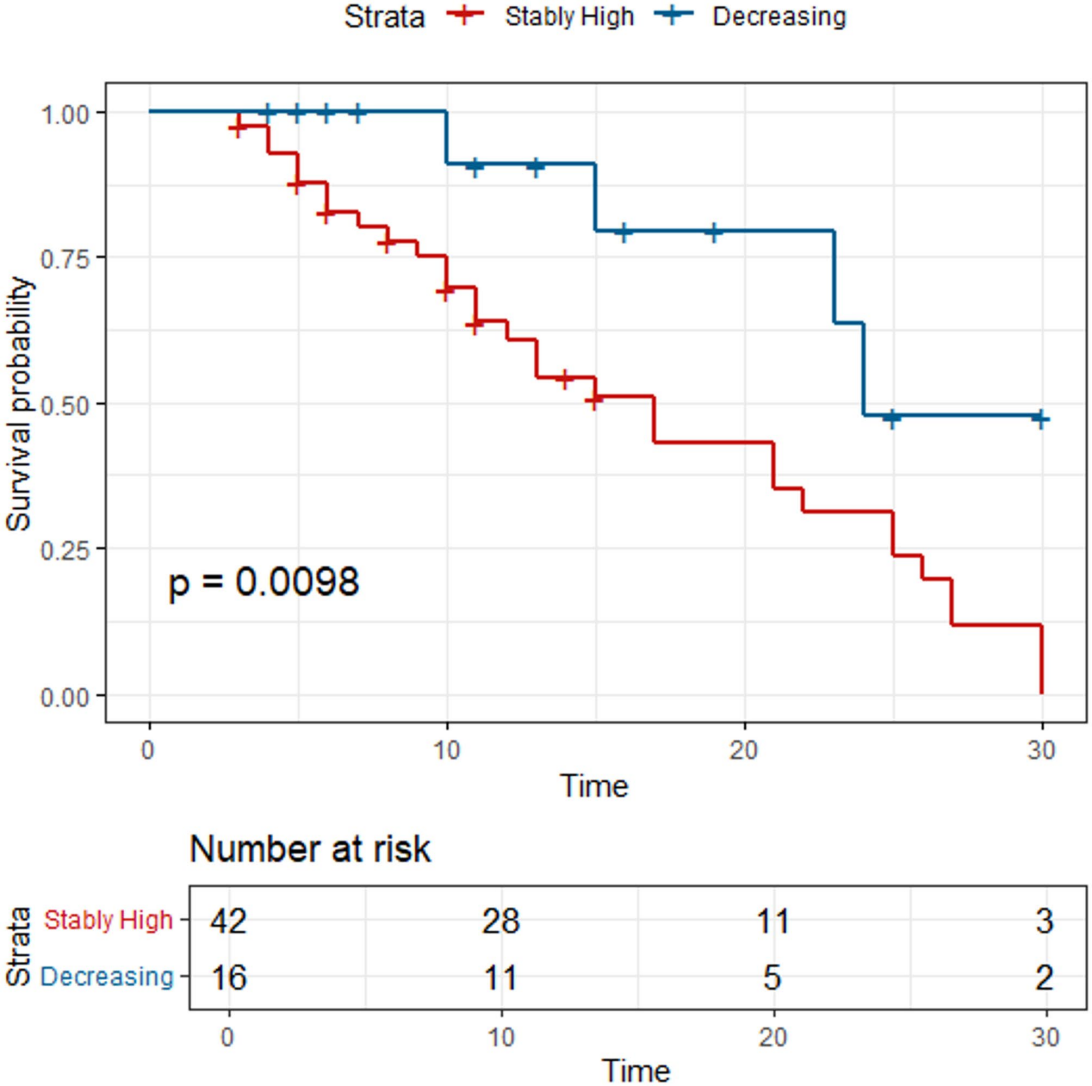
K‒M survival plot of patients with acute kidney injury in the West China Hospital database.

A total of 58 patients were included in the validation cohort. The cohort had a median age of 59 years (IQR 43–66), with 69% of the patients being male. At ICU admission, the median APACHE II score was 19.5 (IQR 14–27). Additional baseline characteristics are detailed in Table 4. The GBTM construction procedure was replicated, and a two-latent-group model was identified as the optimal. Two comparable trajectories were identified and illustrated in Fig. 2. The cohort was stratified into the SH group (*n* = 42, 72.4%) and the D group (*n* = 16, 27.6%). No significant difference in sex distribution was observed between groups (SH vs. D, male sex: 73.8% vs. 56.2%, *p* > 0.05). In contrast to findings from the eICU database, the SH group demonstrated a significantly higher median age compared to the D group (SH vs. D, 60.5 years vs. 48.5 years, *p* = 0.014). Consistent with prior observations, the SH group also exhibited a greater proportion of patients with stage 3 AKI (SH vs. D, 50.0% vs. 31.2%, *p* = 0.042). A comprehensive comparison of baseline characteristics between groups is provided in Table 4. Multivariate Cox proportional hazards analysis revealed that, unlike previous results, subphenotype classification did not emerge as an independent risk factor in either the reduced model (HR 2.03, 95% CI 0.87–4.75, *p* > 0.05, D group as reference) or the full model (HR 1.52, 95% CI 0.54–4.23, *p* > 0.05, D group as reference). However, Kaplan-Meier survival curves (Fig. 5) demonstrated notable divergence between groups, and this visual difference was corroborated by a statistically significant log-rank test (*p* = 0.010).

**Table 4:** Baseline characteristics of the overall population, Decreasing, and stably high groups in West China hospital database. AST: aspartate aminotransferase; ALT: Alanine aminotransferase; WBC: white blood cell count; RBC: red blood cell count; BUN: blood Urea nitrogen; LDH: lactate dehydrogenase; MAP: mean atrial pressure; GCS: Glasgow coma scale; CKD: chronic kidney disease.

	Overall	Decreasing	Stably High	*p*
*N*	58	16	42	
**Age**	59.00 [43.00, 66.00]	48.50 [37.00, 54.50]	60.50 [45.00, 71.00]	0.014
**Sex (%)**				0.330
Female	18 (31.0)	7 (43.8)	11 (26.2)	
Male	40 (69.0)	9 (56.2)	31 (73.8)	
**Hemoglobin**	86.25 [76.83, 100.30]	95.78 [81.11, 111.26]	82.61 [76.53, 93.69]	0.076
**WBC**	11.20 [9.21, 14.16]	10.44 [8.45, 13.73]	11.36 [9.32, 14.40]	0.602
**RBC**	2.81 [2.56, 3.39]	3.10 [2.68, 3.77]	2.78 [2.54, 3.32]	0.122
**Platelets**	156.50 [90.75, 209.50]	163.00 [97.25, 207.25]	152.50 [86.25, 209.50]	0.821
**AST**	62.14 [31.16, 126.19]	48.48 [22.42, 104.02]	77.05 [32.38, 233.14]	0.098
**ALT**	35.33 [18.35, 108.05]	42.87 [14.23, 75.89]	33.48 [21.04, 116.11]	0.602
**Creatinine**	92.00 [59.75, 151.00]	61.50 [52.00, 78.75]	112.00 [73.00, 203.25]	0.007
**BUN**	13.93 [9.41, 19.48]	8.22 [6.79, 15.74]	14.85 [11.45, 20.32]	0.011
**LDH**	357.00 [262.50, 501.00]	389.50 [255.75, 536.00]	340.50 [276.75, 500.00]	0.814
**Spo2**	99.75 [99.39, 99.89]	99.77 [99.57, 99.88]	99.74 [99.31, 99.89]	0.548
**PH**	7.38 [7.35, 7.40]	7.41 [7.38, 7.43]	7.37 [7.34, 7.39]	0.002
**Lactate**	2.09 [1.75, 2.73]	1.76 [1.50, 1.92]	2.33 [1.93, 2.93]	0.002
**HCO3**	19.27 [18.54, 20.19]	19.11 [18.60, 19.69]	19.31 [18.54, 20.30]	0.626
**Sodium**	139.02 [137.35, 141.71]	138.51 [135.09, 139.70]	139.17 [137.55, 142.56]	0.077
**Potassium**	4.13 [3.85, 4.23]	3.75 [3.60, 4.16]	4.19 [4.04, 4.23]	0.012
**Heart Rate**	90.27 [83.30, 97.03]	90.80 [87.13, 97.91]	89.60 [83.30, 96.67]	0.676
**Respiratory Rate**	16.49 [15.15, 18.16]	16.16 [14.53, 16.88]	16.82 [15.42, 19.43]	0.017
**Temperature**	37.09 [36.69, 37.52]	37.18 [36.68, 37.48]	37.09 [36.70, 37.55]	0.676
**MAP**	86.45 [83.01, 89.49]	87.30 [84.50, 93.96]	86.26 [82.29, 89.30]	0.366
**ICU Stay**	11.50 [7.00, 21.00]	14.00 [6.75, 23.25]	11.00 [7.25, 20.00]	0.632
**Clinical Scores**				
GCS	9.00 [5.25, 10.00]	10.00 [8.50, 12.75]	6.50 [4.00, 10.00]	0.004
APACHE	19.50 [14.00, 27.00]	16.00 [10.00, 19.25]	22.00 [15.25, 27.75]	0.024
**Aki grade (%)**				0.042
1	20 (34.5)	9 (56.2)	11 (26.2)	
2	12 (20.7)	2 (12.5)	10 (23.8)	
3	26 (44.8)	5 (31.2)	21 (50.0)	
**Comorbidity (%)**				
Heart failure	16 (27.6)	4 (25.0)	12 (28.6)	1.000
Hypertension	18 (31.0)	3 (18.8)	15 (35.7)	0.352
Diabetes	17 (29.3)	5 (31.2)	12 (28.6)	0.743
CKD	6 (10.3)	1 (6.2)	5 (11.9)	0.881
Shock	36 (62.1)	6 (37.5)	30 (71.4.2)	0.038
Sepsis	16 (27.6)	4 (25.0)	12 (28.6)	1.000

## Discussion

This study identified two clinically distinct AKI subphenotypes—the Stably High (SH) and Decreasing (D) subgroups—characterized by divergent trajectories of EASIX scores during the first seven days of ICU admission in the eICU database. Multivariable Cox proportional hazards analysis demonstrated that subphenotype classification served as an independent risk factor for both 30-day in-ICU mortality and 30-day in-hospital mortality. These findings were subsequently validated in an independent patient cohort from our institution, where the same trajectory patterns emerged. Notably, in this validation cohort, the subphenotypes maintained their significant association with in-ICU mortality, reinforcing the clinical relevance of these distinct phenotypic classifications.

AKI is a rather common yet complicated disease characterized by an increase in serum creatine and/or a decrease in urine output. The condition exhibits substantial etiological and pathophysiological heterogeneity, with traditional classification systems categorizing cases as prerenal, intrinsic renal, or postrenal injury^10,11^. Possible grouping methods include the use of clinical symptoms, serum biomarkers and omics data, such as proteomics and transcriptomics^10^. The KDIGO guidelines utilize creatinine levels and urine outputs to categorize patients, and now serve as the predominant clinical framework. However, several limitations persist in conventional classification systems. The KDIGO criteria, while clinically practical, may introduce population heterogeneity^12^. Even patients within the same AKI group could have various clinical outcomes, as illustrated in recent literature about the second group in KDIGO AKI stage 1^13,14^. While symptom-based stratification shows prognostic value, its utility for therapeutic decision-making remains limited by insufficient mechanistic insight, which may partially explain the negative outcomes in renal replacement therapy trials^15^. Emerging biomarker-based subphenotyping approaches offer promise for addressing these limitations. By capturing distinct pathobiological mechanisms, these methods may enable more precise patient stratification than purely clinical criteria. The integration of biomarker profiling with symptom-based approach could yield a more sophisticated classification paradigm, potentially identifying subgroups amenable to targeted therapeutic interventions. Future research exploring this combined approach may significantly advance AKI management strategies.

The pursuit of refined AKI classification represents a critical step toward personalized medicine in nephrology. By moving beyond traditional categorization systems, researchers may identify more homogeneous patient populations that better reflect underlying pathophysiology. While multi-omics approaches theoretically offer the highest resolution for subphenotyping, practical constraints including substantial costs and limited sample availability currently restrict their widespread clinical application. Alternative approaches using routinely available clinical data have shown promise, as demonstrated by Bhatraju et al.‘s creatinine trajectory analysis in ICU populations^3^. Their binary classification of resolving versus non-resolving AKI based on serum creatinine changes (≥ 0.3 mg/dL or 25% decrease within 72 h) revealed meaningful risk stratification, with non-resolving cases showing an RR of 1.52 (95% CI 1.13–2.05) compared to non-AKI controls. Notably, their findings also showed limited prognostic value in conventional KDIGO staging, suggesting the need for alternative classification paradigms. However, this creatinine-focused approach has inherent limitations including potential overestimation of effect sizes from p-value driven selection and reduced discriminative capacity evidenced by overlapping risk estimates between Resolving and Non-resolving AKI groups. More fundamentally, the univariate nature of creatinine trajectory analysis fails to capture the multidimensional complexity of AKI pathophysiology. In this context, our GBTM approach offers distinct advantages through its incorporation of multiple clinical variables into a composite index, providing both richer trajectory interpretation and enhanced phenotypic discrimination. This multivariate method better accommodates the biological and clinical heterogeneity of AKI while remaining feasible for both research and clinical applications, potentially serving as a bridge toward more comprehensive omics-based classification systems as they become more accessible.

The EASIX score, originally developed by Luft et al. for mortality prediction in graft-versus-host disease following allogeneic stem-cell transplantation^16^. We chose EASIX as the foundation for our trajectory analysis due to several compelling advantages. Its biological relevance to AKI pathophysiology stands as the primary rationale, with demonstrated capacity to reflect thrombotic microangiopathy, which is a key mechanistic component in AKI initiation and progression^17^. This characteristic suggests EASIX trajectories may capture pathophysiological nuances that conventional biomarkers like creatinine and blood urea nitrogen fail to reveal. From a practical standpoint, EASIX offers significant clinical utility as its components (lactate dehydrogenase, creatinine, and platelet counts) are routinely measured in ICU settings, ensuring both accessibility and longitudinal traceability. The score’s objectivity provides another distinct advantage over many comprehensive clinical scoring systems. Unlike assessments requiring subjective interpretation such as the Glasgow Coma Scale, EASIX derives entirely from standardized laboratory parameters, eliminating inter-rater variability and enhancing reproducibility across clinical environments^18^.

GBTMs has emerged as a powerful analytical approach for disease subphenotyping across various clinical contexts. The method’s growing adoption stems from its unique ability to capture disease evolution through longitudinal patterns, as demonstrated in Xu et al.‘s recent application to sepsis subphenotyping using sequential organ failure assessment score trajectories^19^. At its core, GBTM operates on the principle that observed clinical data represent a finite mixture of latent subpopulations, each with distinct trajectory patterns^20^. This approach offers several key advantages over traditional classification methods. GBTM utilizing longitudinal data to model disease progression dynamics incorporates substantially more clinical information than single-timepoint assessments. Flexible distributional requirements of linear mixed models accommodate real-world clinical data that often violates conventional statistical assumptions. More importantly, GBTM provides probabilistic classification, explicitly quantifying the uncertainty of subgroup membership, which is a feature that enhances both the methodological rigor and clinical interpretability of the results^20^.

However, Cox regression analysis of data from the West China Hospital database indicated an HR of 2.03 (95% CI 0.87–4.75) in the reduced model and 1.52 (95% CI 0.54–4.23) in the full model. Possible reasons for this inconsistent result may arise from the following factors. Most notably, the relatively small validation cohort (*n* = 58 AKI patients) may have limited statistical power for robust Cox model estimation, particularly when adjusting for multiple covariates in the full model. Interestingly, despite these null findings in the regression analysis, the nonparametric log-rank test demonstrated statistically significant separation between SH and D group survival curves (*p* = 0.010, Fig. 5), suggesting the potential clinical relevance of these trajectory-based subphenotypes may persist even in smaller samples. To better examine the validity of these findings, larger external cohorts are needed.

This study makes several important contributions to AKI phenotyping by use of longitudinal EASIX trajectories during the first week of ICU stay. By moving beyond traditional single-timepoint assessments, we identified two distinct AKI subphenotypes characterized by divergent clinical trajectories, each demonstrating unique patterns of laboratory abnormalities, comorbid conditions, and AKI severity. The external validation using data from West China Hospital significantly strengthens these findings by demonstrating consistent trajectory patterns across independent patient populations. These results not only provide new insights into potential mechanistic differences underlying AKI progression but also open promising avenues for personalized treatment approaches. Of particular clinical relevance is the potential for these subphenotypes to exhibit differential responses to renal replacement modalities, suggesting future research should focus on optimizing therapy selection based on early trajectory patterns.

Several important limitations warrant consideration when interpreting these findings. The retrospective design requires cautious interpretation of the observed associations. Data constraints presented additional challenges, notably the limited availability of lactate dehydrogenase measurements in the eICU database (2014–2015 cohort). However, our final analytic cohort of 317 patients met conventional sample size requirements^20^. The West China Hospital validation may have underestimated true AKI prevalence due to unavailable pre-ICU clinical and laboratory records, potentially excluding cases diagnosed immediately before ICU transfer. The exclusive inclusion of critically ill patients restricts generalizability to other clinical environments, while the inability to distinguish community-acquired from hospital-acquired AKI (due to missing presentation histories) represents another important constraint. This particular limitation highlights a valuable opportunity for future prospective studies to better characterize acquisition settings and their potential influence on trajectory patterns.

## Conclusion

Our analysis of EASIX trajectories in ICU-admitted AKI patients revealed two clinically distinct subphenotypes characterized by significant differences in presentation, laboratory profiles, comorbid conditions, and outcomes, which strongly suggests divergent pathophysiological pathways. Further investigations investigating the early prediction of the two subphenotypes, estimation of different treatment responses and elucidation of the molecular mechanisms underlying the differences between these two groups can be performed. The trajectory-based phenotyping approach demonstrated here offers a dynamic framework that captures disease evolution more effectively than traditional static assessments, potentially enabling more personalized management strategies for critically ill AKI patients.

## Data Availability

Data from the eICU collaborative research database are available in the eICU repository (https://physionet.org/content/eicu-crd/2.0/). The data from West China Hospital are available from the corresponding authors upon reasonable request.

## Abbreviations

AKI: Acute kidney injury
ICUs: Intensive care units
KDIGO: The Kidney Disease: Improving Global Outcomes
EASIX: Endothelial activation and stress index
GBTM: Group-based trajectories modeling
HR: Hazard Ratio
APACHE: Acute Physiology And Chronic Health Evaluation
